# A new species of Fuziidae (Insecta, Blattida) from the Inner Mongolia, China

**DOI:** 10.3897/zookeys.217.3508

**Published:** 2012-08-28

**Authors:** Dandan Wei, Junhui Liang, Dong Ren

**Affiliations:** 1 Key Lab of Insect Evolution and Environmental Changes,; 2 Capital Normal University, Beijing 100048, China; 3 Tianjin Museum of Natural History, 206 Machang Road, Hexi District, Tianjin, 300074, China

**Keywords:** Cockroach, Blattida, Fuziidae, new species, Middle Jurassic, Daohugou, China

## Abstract

A new species attributed to the genus *Parvifuzia* Guo & Ren, 2011, *Parvifuzia peregrina* **sp. n.**, is described from the Middle Jurassic Jiulongshan Formation of Daohugou Village, Inner Mongolia, China. This new species, with apex of wing almost reaching the end of the abdomen and forewing venation with 30–32 veins at margin, broadens the diversity of *Parvifuzia*. This new species, with strongly curved cerci, could tightly clasp female and complete copulation more efficiently, same as other members of the family Fuziidae.

## Introduction

Fuziidae, a small extinct family, was erected by Vršanský et al. (2009). Until now, only three genera and four species have been described: *Fuzia dadao* Vršanský, Liang & Ren, 2009, *Parvifuzia marsa* Guo & Ren, 2011, *Parvifuzia brava* Guo & Ren, 2011 and *Colorifuzia agenora* Wei, Liang & Ren (Vršanský et al. 2009; Guo and Ren 2011; Wei et al. in press).

This family is only found in the Middle Jurassic of China so far. It might have originated during the Triassic and became extinct in the Late Jurassic (Vršanský et al. 2009). This family is distinguished by its unique structure of male’s elongated body and forceps of earwig-like cerci (attached to the external ovipositor during courtship).

Recently we collected three well-preserved fossils of Fuziidae from Jiulongshan Formation in Daohugou Village, Ningcheng County, Inner Mongolia, China. After detailed examination, we consider these specimens belonging to the genus *Parvifuzia* based on the following features: small-sized (body length 10.4–10.6 mm and forewing length 8.5–8.8 mm); apex of cerci strongly curved inward, with a narrow gap at the center; wing venation simple; forewing R strongly curved, CuA almost straight, then curved to posterior wing margin.

Daohugou has yielded many fossil insects, animals and plants. Many insects have been found in this biota, such as Orthoptera (Gu et al. 2012), Grylloblattida (Cui et al. 2010), Mecoptera (Ren et al. 2009), Neuroptera (Wang et al. 2010), Plecoptera (Liu et al. 2008), Hemiptera (Yao et al. 2007), Hymenoptera (Shih et al. 2011), Diptera (Zhang et al. 2009), carnivorous cockroaches (Liang et al. 2009) and Ephemeroptera (Huang et al. 2008). The age of Jiulongshan Formation, although still controversial, is generally accepted as the Middle Jurassic (Bathonian-Callovian boundary interval, about 165 Mya) (Ren et al. 2002; Chen et al. 2004; Gao et al. 2006).

## Material and methods

Three specimens of the new species are deposited in the fossil insect collection of the Key Laboratory of Insect Evolution & Environmental Changes, Capital Normal University, Beijing, China. The specimens were examined with a Leica MZ 12.5 dissecting microscope and illustrated with the aid of a drawing tube attached to the microscope. Line drawings were made with Photoshop CS 8.0 graphic software. Photographs of fossils were taken by a MZ12.5 dissecting microscope (Leica, Wetzlar, Germany), either dry or with alcohol.

The venation nomenclature used in this paper is based on the interpretation of Vršanský (1997, 2009). Abbreviations used: RFW- Right forewing; LFW- Left forewing; HW- Hind wing; Sc- Subcosta; R- Radius; Rs- Radius Sector; M- Media; Cu- Cubitus (A- anterior, P- posterior); A- Anal vein; Ant- Antenna.

## Systematic palaeontology

### Order Blattida Latreille, 1810. Superfamily Caloblattinoidea Vršanský & Ansorge in Vršanský (2000). Family Fuziidae Vršanský, Liang & Ren, 2009

#### Parvifuzia

Guo & Ren, 2011

http://species-id.net/wiki/Parvifuzia

##### Type species.

*Parvifuzia marsa* Guo & Ren, 2011

##### Other species included.

*Parvifuzia brava* Guo & Ren, 2011, and *Parvifuzia peregrina* sp. n.

##### Key to species of the genus *Parvifuzia*

**Table:** 

1	Apex of wing reaching the middle of the 8^th^ abdominal segment	2
–	Apex of wing almost reaching the end of the abdomen	*Parvifuzia peregrina* sp. n.
2	Pronotum quasi-circular, not very wide; forewing M reaching the anterior margin of the wing	*Parvifuzia marsa* Guo & Ren, 2011
–	Pronotum wide, oval, transverse; forewing M reaching the apex margin of the wing	*Parvifuzia brava* Guo & Ren, 2011

#### Parvifuzia peregrina

Wei, Liang & Ren sp. n.

urn:lsid:zoobank.org:act:64133161-19F9-41EB-A9DD-0D5B94F65A52

http://species-id.net/wiki/Parvifuzia_peregrina

Figs 1 [Fig F2] –3

##### Diagnosis.

Apex of wing almost reaching the end of the abdomen, forewing venation with 30–32 veins at margin.

##### Comments.

*Parvifuzia peregrin*a sp. n. is similar to *Parvifuzia marsa* Guo & Ren, 2011 in the following aspects: small-sized; apex of cerci strongly curved inward and rounded in shape, with a narrow gap at the center; wing venation simple; forewing R strongly curved like waves, CuA almost straight, then curved to posterior wing margin, anal area wide.

However, *Parvifuzia peregrin*a sp. n. can be easily differentiated from the other two previously described species: apex of wing almost reaching the end of the abdomen in *Parvifuzia peregrin*a sp. n. vs. apex of wing just reaching the middle of the 8^th^ abdominal segment; forewing length is longer (forewing length 8.5–8.8 mm in *Parvifuzia peregrin*a sp. n., vs. forewing length 6.3–6.4 mm); and forewing venation with 30–32 veins at margin in *Parvifuzia peregrina* sp. n., vs. forewing venation with 25–27 veins at margin.

##### Description.

Small-sized, body length about 10.4–10.6 mm (with head), width 2.8–3.1 mm; head small, significantly elongated (length/width= 1.4–1.6 mm/1.3–1.4 mm), antennal socket conspicuous at sides, mouthparts unclear; pronotum length 1.6–1.9 mm, width 2.3–2.7 mm, elliptical, as wide as the body; abdomen 6–7 segments visible, terminal sternum rounded; long cerci has 14 segments and apex of cerci strongly curved inward and rounded in shape, forming a narrow gap at center (Fig. 3A), segments of cerci joined together after the 8^th^ segment.

**Figure 1.**
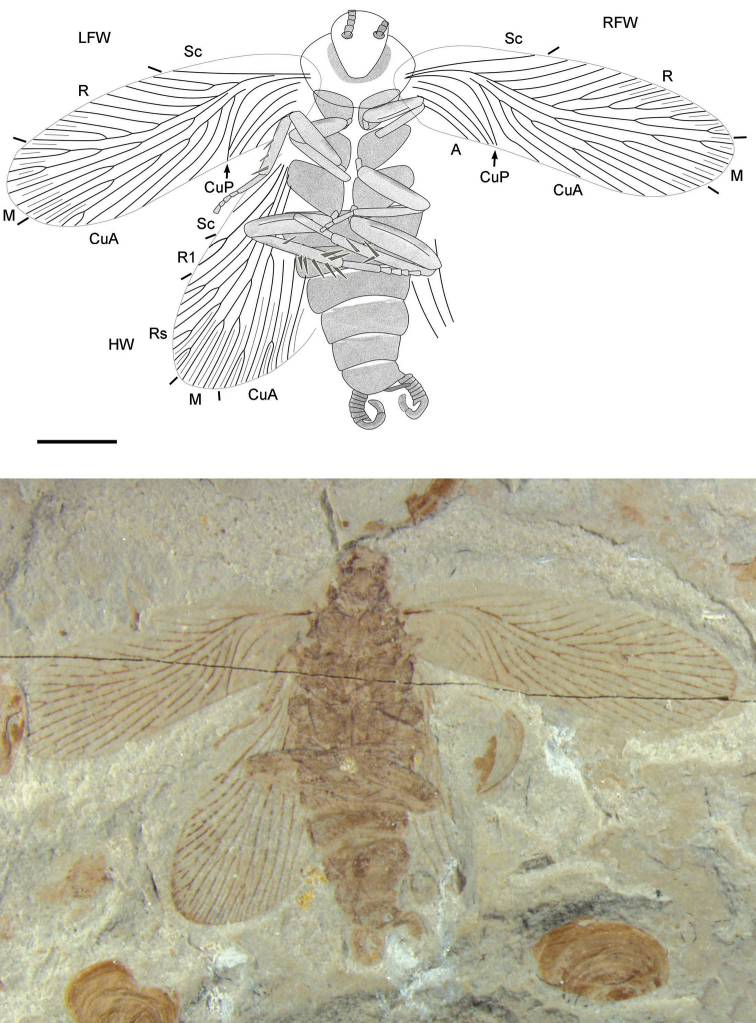
*Parvifuzia peregrina* sp. n. Holotype, CNU-BLA-NN-2011055; Line drawing and photograph. Scale bar = 2 mm.

**Figure 2. F2:**
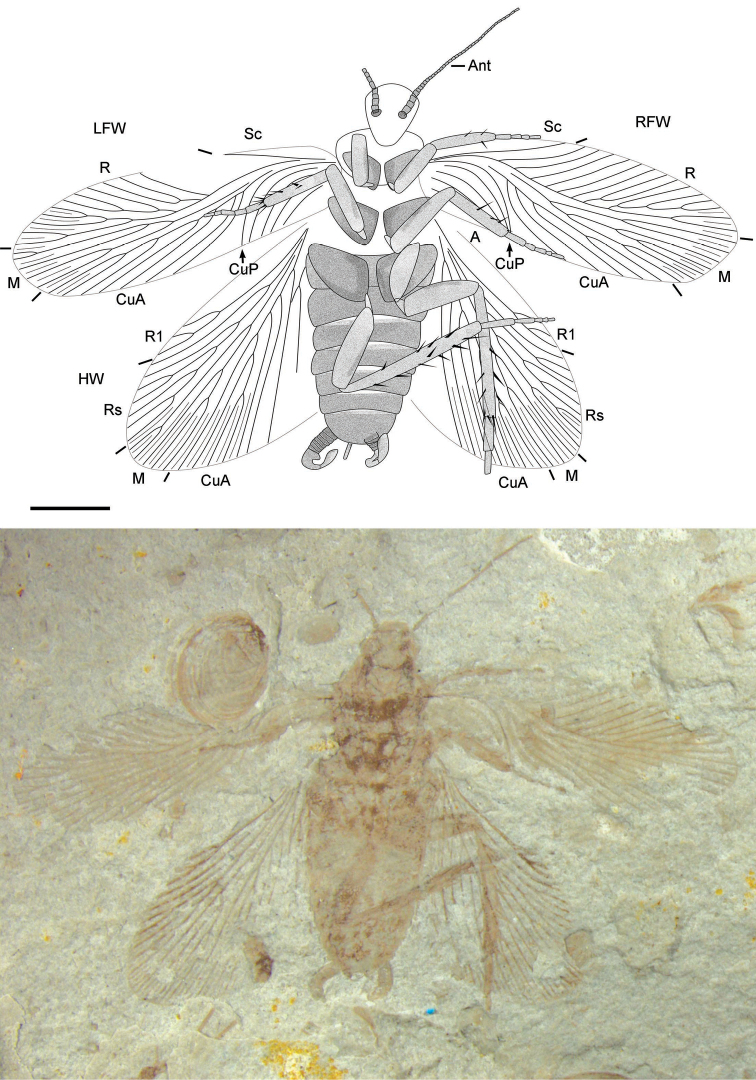
*Parvifuzia peregrina* sp. n. Paratype, CNU-BLA-NN-2011057; Line drawing and photograph. Scale bar = 2 mm.

**Figure 3.**
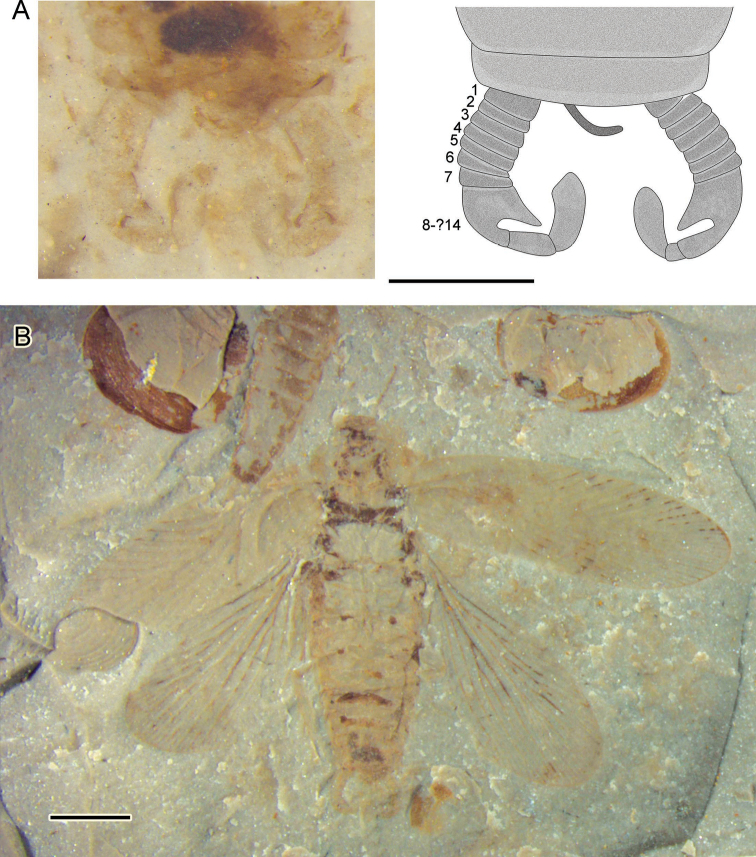
*Parvifuzia peregrina* sp. n. Paratype, CNU-BLA-NN-2011056 **A** Line drawing and photograph of detail of male paratype terminalia with forceps-like cerci. Scale bar = 1 mm **B** photograph. Scale bar = 2 mm.

Forewings (Figs 1, 2, 3B): length range about 8.5–8.8 mm, width range about 2.6–2.9 mm; narrow, without coloration, with intercalaries and wing venation simple, with 30–32 veins at margin; costal area wide (1/3 width of the wing); Sc simple, curved upward, longer than clavus; R strongly curved like waves and with 9–14 branches, reaching the anterior wing margin; M slightly curved and with 5–7 branches, most posterior branch of M reaching wing apex; CuA almost straight to posterior wing margin and with 5–8 branches; CuP strongly curved and simple; clavus short, less than a third of the wing’s length; A simple, arc bending and with about 4 veins.

Hind wings: length about 6.5–7.1 mm, width of remigium 2.8–3.5 mm; with intercalaries and without pterostigma; with about 22 veins of remigium; Sc simple, sometimes unclear; R terminating to wing apex, differentiated into darkened R1 with 2–3 branches and Rs with 7–9 branches; M almost straight to posterior wing margin, with 3–5 branches; CuA with about 7 branches.

Legs: length of fore femora 1.16–1.49 mm and tibiae 1.16–1.22 mm, length of mid femora 1.89–1.93 mm and tibiae 1.47–1.56 mm, length of hind femora 2.12–2.19 mm and tibiae 2.59–3.45 mm; legs gradually get longer from the front to the hind legs; mid and hind leg with spines on the tibiae.

##### Materials.

Holotype, A completely preserved male specimen, CNU-BLA-NN-2011055. Paratypes, CNU-BLA-NN-2011056, CNU-BLA-NN-2011057.

##### Type locality and horizon.

Jiulongshan Formation; Daohugou Village, Wuhua Township, Ningcheng County, Inner Mongolia, China; Middle Jurassic.

##### Etymology.

The specific name is derived from the Latin word “*peregrinus*”, (meaning “strange”), for this new species is special for specific characters.

## Discussion

As shown in Table 1, although the total number of forewing veins of *Parvifuzia peregrina* is stable, variability of the wing venation and difference between the left and right wings of the same individual are obvious.For example, the right forewing of holotype of *Parvifuzia peregrina* differs from the left forewing in the following characters: (1) R with 9 branches in right forewing vs. 13 branches in left; (2) M with 7 branches in right forewing vs. 5 branches in left; (3) CuA with 8 branches in right forewing vs. 7 branches in left. We can also see the difference between the left and right wings of the paratype of *Parvifuzia peregrina*.

**Table 1.** Variability of forewing venation for *Parvifuzia*.<br/>

**Species**	**Number**	**Right forewing**							**Left forewing**						
		**Sc**	**R**	**M**	**CuA**	**CuP**	**A**	**Total**	**Sc**	**R**	**M**	**CuA**	**CuP**	**A**	**Tota**
*Parvifuzia marsa*	CNU-BLA-NN-2009030	1	10	6	6	1	3	27	1	11	4	6	1	3	26
*Parvifuzia brava*	CNU-BLA-NN-2009031	1	12	3	5	1	3	25							
*Parvifuzia peregrina*	CNU-BLA-NN-2011055	1	9	7	8	1	4	30	1	13	5	7	1	4	31
	CNU-BLA-NN-2011057	1	11+	5	7	1	4	29+	1	14	7	5	1	4	32

## Supplementary Material

XML Treatment for Parvifuzia

XML Treatment for Parvifuzia peregrina
